# Assessment of the Sustainability of the Mediterranean Diet Combined with Organic Food Consumption: An Individual Behaviour Approach

**DOI:** 10.3390/nu9010061

**Published:** 2017-01-12

**Authors:** Louise Seconda, Julia Baudry, Benjamin Allès, Oualid Hamza, Christine Boizot-Szantai, Louis-Georges Soler, Pilar Galan, Serge Hercberg, Denis Lairon, Emmanuelle Kesse-Guyot

**Affiliations:** 1 Equipe de Recherche en Epidémiologie Nutritionnelle (EREN), Université Paris 13, Inserm (U1153), Inra (U1125), Centre d’Epidémiologie et Statistiques Paris Cité, Cnam, COMUE Sorbonne-Paris-Cité, 93017 Bobigny, France; j.baudry@eren.smbh.univ-paris13.fr (J.B.); b.alles@eren.smbh.univ-paris13.fr (B.A.); p.galan@uren.smbh.univ-paris13.fr (P.G.); s.hercberg@uren.smbh.univ-paris13.fr (S.H.); e.kesse@eren.smbh.univ-paris13.fr (E.K.-G.); 2 INRA Aliss UR 1303, 94200 Ivry sur Seine, France; Oualid.Hamza@ivry.inra.fr (O.H.); Christine.Boizot@ivry.inra.fr (C.B.-S.); lgsoler@ivry.inra.fr (L.-G.S.); 3 Département de Santé Publique, Hôpital Avicenne, 93017 Bobigny, France; 4 Nutrition, Obésité et Risque Thrombotique (NORT), Aix Marseille Université, INSERM, UMR S 1062, INRA 1260, 13005 Marseille, France; denis.lairon@orange.fr

**Keywords:** Mediterranean diet, organic diet, sustainability indicators, individual behaviours, nutrition, economy, environmental impact

## Abstract

Mediterranean diets are promising sustainable food models and the organic food system may provide health and environmental benefits. Combining the two models could therefore be a favourable approach for food sustainability. The aim of this study was to draw up a comparative description of four diets differing in the level of organic foods consumption and the adherence to the Mediterranean diet, using multidisciplinary indicators to assess the sustainability of these diets. Four groups of participants were defined and compared, combining the proportion of organic food in their diet (Org versus Conv) and the adherence to the Mediterranean diet (Med versus NoMed). Conv–NoMed: Conventional consumers and non-Mediterranean diet followers; Conv–Med: Conventional consumers and Mediterranean diet followers; Org–NoMed: Organic consumers and non-Mediterranean diet followers; Org–Med: Organic consumers and Mediterranean diet followers. The adherence to nutritional recommendations was higher among the Org–Med and Conv–Med groups compared to the Conv–NoMed group (using the mPNNS-GS (modified-Programme National nutrition santé guidelines score/13.5 points): 9.29 (95% confidence intervals (CI) = 9.23–9.36) and 9.30 (95% CI = 9.24–9.35) versus 8.19 (95% CI = 8.17–8.22)) respectively. The mean plant/animal protein intake ratio was 1.38 (95% CI = 1.01–1.74) for the Org–Med group versus 0.44 (95% CI = 0.28–0.60) for the Conv–NoMed group. The average cost of the diet of Org–Med participants was the highest: 11.43 €/day (95% CI = 11.34–11.52). This study highlighted the importance of promoting the Mediterranean diet combined with organic food consumption for individual health and environmental aspects but challenges with regard to the cost remain.

## 1. Introduction

It is established that the current food systems cause major pressure on the environment [1] and impact human health [2]. In particular, the westernization of human diets is an important factor for the development of some chronic diseases [3]. In a context of a noticeable growth of the world population, adequately feeding more than 9 billion people by 2050 remains a major challenge. In particular, the increase in the demand for animal proteins is a critical issue because large-scale livestock production considerably impacts the environment (to produce 1 kg of edible beef protein, 20 kg equivalent of plant proteins are required [4]).

Therefore, the challenge of offering appropriate feeding to the global world population could not be solved without embedding a dietary pattern perspective that includes preservation of the human health and planet resources [5]. For that purpose, the FAO (Food and Agriculture Organization of the United Nations) provided a definition of sustainable diets as those “with low environmental impacts which contribute to food and nutrition security and to healthy life for present and future generations. They are protective and respectful of biodiversity and ecosystems, culturally acceptable, accessible, economically fair and affordable; nutritionally adequate, safe and healthy; while optimizing natural and human resources” [6].

For decades, the Mediterranean diet has been considered as a paradigm of healthy diets [7]. Indeed, high adherence to the Mediterranean diet is protective against the occurrence of overall mortality, incidence and mortality from cardiovascular diseases and cancers [8]. Moreover, Mediterranean diets may better suit the concept of sustainable diets as they imply a limitation of meat intake [9,10]. Additionally, the Mediterranean food system is also a universal, cultural, social and spatial heritage of all civilizations living around the Mediterranean basin registered by the UNESCO (United Nations Educational, Scientific and Cultural Organization) as an immaterial human heritage (including practices, representations, expressions, knowledge, skills as well as the instruments, objects, artefacts and cultural spaces associated there with, that communities, groups and, in some cases, individuals recognize as part of their cultural heritage) in 2010. Unfortunately, this precious heritage is being progressively abandoned [7,11,12].

Besides, compared to conventional farming, organic farming has been shown to be a potentially more environmentally friendly food production system [1,13]. Although studies directly investigating the role of organic food consumption on health are scant, it has been shown that organic food consumers exhibit heathier dietary patterns [13,14].

In that context, organic food consumption and Mediterranean diets can be seen as promising models of sustainable diets. However, to our knowledge, no study has evaluated these dietary behaviours, alone or in combination, in terms of sustainability-related characteristics using individual behaviours.

The objective of the present study was thus to describe and compare four different subgroups of subjects based on their level of consumption of organic food and their adherence to the Mediterranean diet using sociocultural, economic, nutritional and environmental factors as indicators of the sustainability of the diet, as proposed by the FAO [6], in a large sample of participants from the NutriNet-Santé study.

## 2. Materials and Methods

### 2.1. Participants

Subjects were participants of the NutriNet-Santé Study, an ongoing web-based prospective observational French cohort of volunteers aged 18 years and older, launched in May 2009 through recruitment planned over a 5-year period. The design of the NutriNet-Santé Study has been described in detail elsewhere [15]. Briefly, to be included in the cohort, participants had to fill in self-administered web-questionnaires providing information on sociodemographic, anthropometric and lifestyle characteristics as well as dietary intake (assessed by repeated 24 h records) along with health status. As part of their follow-up, participants are regularly invited to update the same set of questionnaires. They are also invited to fill in optional complementary questionnaires.

### 2.2. Data Collection

#### 2.2.1. Socio-Demographic and Lifestyle Characteristics

Sociodemographic and lifestyle data included age; sex; education level (lower than high school diploma, high school and post-secondary graduate); monthly income (<1200 euros, between 1200 and 1800 euros, between 1800 and 2700 euros and >2700 euros per household unit); place of residence (rural community, urban units with a population smaller than 20,000 inhabitants, between 20,000 and 200,000 inhabitants and higher than 200,000 inhabitants); practice of a specific diet (vegetarian, and vegan diets); smoking status (former, occasional, current or non-smoker); level of physical activity (as measured by the IPAQ (International Physical Activity Questionnaires) [16,17,18]); household size; number of children; and income. Income per month and per household unit was obtained by dividing income by the number of consumption units (CU): 1 CU for the first adult in the household, 0.5 CU for other persons older than 14 years old and 0.3 CU for others. Participants were asked to report weight and height assessed by a health worker, physician or from self-measurement guided by standardized procedures. Body mass index (BMI) (kg/m^2^) was calculated at enrolment and yearly.

In 2014, they were also asked to complete an optional questionnaire focusing on attitudes and motivations for food purchases. The questionnaire included questions pertaining to places of food purchase, as well as sustainable-related motivations when purchasing (including seasonality, geographical origin of foods, farming production methods, ethics, contact with producers and regional and traditional foods).

#### 2.2.2. Assessment and Treatment of Dietary Data

In July 2014, participants were asked to fill in an organic food semi-quantitative frequency questionnaire (Org-FFQ) [14], based on a previously validated FFQ [19]. In the Org-FFQ, participants were asked to report their consumption frequencies (yearly, monthly, weekly or daily units) and their portion size (described as typical household measurements or with colour photographs) for 264 food and beverage items over the past year. In order to estimate total food intake, the portion size was multiplied by its frequency.

To assess organic food consumption, participants were also asked, for each food item, to report their frequency of consumption in its organic form through a 5-point ordinal scale ranging from “never” to “always”. Weightings of 0, 0.25, 0.5, 0.75 and 1 were allocated to the corresponding modalities: never, rarely, half of time, often and always. This enabled us to calculate, at an individual level, the share of organic food in the diet for each food. Nutrients intakes were estimated using the published NutriNet-Santé food composition database [20]. To depict the nutritional quality of the diet, three a priori dietary scores were computed: the PANDiet (a diet quality index based on the Probability of Adequate Nutrient intake) [21], the mPNNS-GS (modified Programme National Nutrition Santé-Guidelines Score) [22] and a dietary diversity score [23] (Table 1). Energy density, defined as the energy intake per 100 g of the total intake, was also computed.

#### 2.2.3. Assessment of the Cost of the Diet

The individual daily cost of the diet was assessed using a specifically developed price database. A cost was assigned to each food item taking into account the place of purchase and the mode of food production (organic vs. conventional). For food purchased from supermarkets and specialized stores, the 2012 KANTAR database was used [24]. Additionally, to enable allocation of food prices purchased in short supply chains (local markets or associations supporting small farming (AMAPs)) 1100 prices were collected in autumn 2014 and 862 prices in spring 2015 over nine French metropolitan departments by members of the Bioconsom’acteurs association using a standardized procedure. Finally, we assessed the share of the budget allocated to foods by dividing the total diet cost by the income reported by the participants. As incomes were declared in categories, the mean of each class was used and arbitrary incomes were determined for extreme classes.

### 2.3. Data Computation and Statistical Analyses

Among the 33,384 participants who filled in the Org-FFQ, we excluded those who were under reporting or over reporting (*N* = 2097). Energy requirement, accounting for physical activity level and basal metabolic rate, estimated by Schofield’s [25] equations according to sex, body mass index and age, was compared with energy intake for each individual. The ratio energy intake divided by energy requirement was calculated. Individuals with a ratio below or above cut-offs (0.35 and 1.93) were excluded. We also excluded participants with missing socio-demographic or economic data as well as those who did not complete the optional questionnaire. The final sample included 22,866 participants (16,775 women and 6091 men).

The proportion of organic food consumption in the diet was calculated by dividing the total organic food intake (g/day) by the total intake (g/day) excluding water. An organic consumer was defined herein as a person with a proportion of organic food in the diet at least equal to 50%.

The literature-based adherence score to the Mediterranean diet created by Sofi et al. was computed [8] (Table 1). Following the Mediterranean diet was defined as having a score at least equal to 11 (corresponding to the fourth quartile value). Using the two parameters described above i.e., (1) proportion of organic food in the diet and (2) adherence to the Mediterranean diet, four subgroups were considered. The first group, named Conv–NoMed was composed of subjects with CONVentional consumption who did not follow the MEDiterranean diet. The second group, named Conv–Med included subjects with CONVentional consumption and following the MEDiterranean diet, the third, named Org–NoMed, was composed of subjects with ORGanic consumption who did not follow the MEDiterranean diet, and the fourth, named Org–Med, included subjects with ORGanic consumption and following the MEDiterranean diet.

The sustainability of the four group diets (Conv–NoMed, Org–NoMed, Conv–Med and Org–Med) was estimated using indicators of the nutritional quality of the diet (the PANDiet [21], the mPNNS-GS [22], the dietary diversity score [23], the energy density and the BMI), economic indicators (daily diet cost, total cost for 100 g diet, cost of 100 organic or 100 conventional calories intake), and proxies of environmental indicators (plant/animal protein intake ratio, and source of intake of animal proteins [26]). Protein intake was energy-adjusted using the residual method [27]. We also assessed the importance of sustainability-related motivations when purchasing foods in each group.

Socio-demographic and lifestyle characteristics of the sample were presented across groups; values were stated as means with 95% confidence intervals (CI) or percentages. For descriptive variables, overall differences were tested using χ^2^ or Kruskal–Wallis tests.

Adjusted means or adjusted percentages and 95% CI were provided for each indicator. Adjusted differences between groups (Conv–NoMed, Org–NoMed, Conv–Med and Org–Med) for each indicator were assessed using analysis of covariance (ANCOVA) for continuous variables and logistic regression for qualitative variables. Post-hoc differences in adjusted means across categories were evaluated after adjustment for multiple testing using the Bonferroni’s correction. For statistical tests, the type I error was set at 5%.

All analyses were performed using SAS 9.4 software (SAS Institute Inc., Cary, NC, USA).

## 3. Results

The sizes of the four groups obtained were as follows: Conv–NoMed, *N* = 14,266 (62%) participants; Conv–Med, *N* = 3498 (15%) participants; Org–NoMed, *N* = 2532 (11%) participants and Org–Med, *N* = 2570 (12%) participants.

### 3.1. Characteristics of the Participants

Table 2 shows the main characteristics of the study sample. Participants who were high organic food consumers, were often also better Mediterranean diet followers, and vice versa. Mediterranean diet score was 7.55 (95% CI = 7.52–7.57) among the Conv–NoMed group and 8.39 (95% CI = 8.33–8.46) among the Org–NoMed group respectively, and the mean proportion of organic food of the Conv–Med group and the Conv–NoMed group were 22.4 (95% CI = 21.9–22.9) and 15.5 (95% CI = 15.3–15.8), respectively.

Participants following the Mediterranean diet (Conv–Med and Org–Med) were the oldest while the highest percentages of men and participants with a low educational level were found in ‘Conventional’ groups. Smokers and inactive participants were more likely to be in the Conv–NoMed group, while never smokers, active participants and vegetarians were more frequently observed in the Org–Med group. Low alcohol drinkers were more frequently found in the Org–NoMed or Org–Med groups.

Of note, the overall distribution of the income between groups was different (*p* < 0.0001). However, when we specifically compared the distribution using adjustment for multiple testing via the Bonferroni’s correction, percentages became statistically similar across dietary groups (data not tabulated).

### 3.2. Diet Quality and Dietary Diversity

Table 3 presents the results concerning nutrition-related indicators. Participants in the Org–Med group were those with the lowest BMI. The BMI of Conv–NoMed participants were on average almost 2 points higher compared to those in the Org–Med group. The average BMI of participants in the Conv–Med or Org–NoMed groups were intermediate and comparable.

Participants following the Mediterranean diet (Conv–Med and Org–Med) had higher PANDiet and mPNNS-GS scores as well as a higher diversity score. The Org–Med group exhibited also the highest PANDiet score. The Conv–NoMed group exhibited the lowest scores for PANDiet and mPNNS-GS as well as a higher energy density compared to other groups.

### 3.3. Plant and Animal Protein Source Intake as a Proxy for Environmental Impact

As shown in Table 3, participants in the Org–Med group exhibited a lower protein intake. Their plant/animal proteins intake ratio was 1.38 (95% CI = 1.01–1.74) while it was 0.44 (95% CI = 0.28–0.60) among subjects of the Conv–NoMed group. The intakes of total proteins in the Org–NoMed and Conv–Med groups were comparable. However, participants in the Conv–Med group had higher intakes of plant proteins than those belonging to the Org–NoMed group.

**Figure 1:**
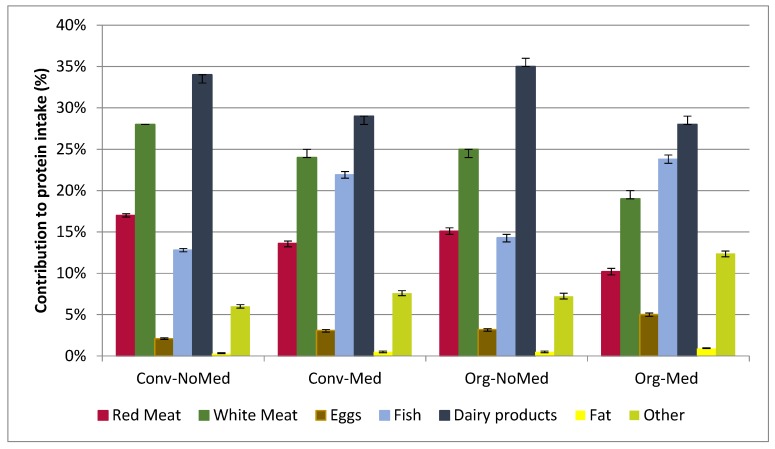
Contribution of food groups to animal protein intake (%). Red Meat: beef, veal and lamb; White meat: pork and poultry; Eggs: eggs; Fish: fish and seafood; Dairy products: milk, cheese, yogurt and other milk-based desserts; fat: butter, sauce, cream; Other foods: chocolate, candy and composite dishes. Conv–NoMed: Conventional consumers and non-Mediterranean diet followers; Conv–Med: Conventional consumers and Mediterranean diet followers; Org–NoMed: Organic consumers and non-Mediterranean diet followers; Org–Med: Organic consumers and Mediterranean diet followers. Values are adjusted for sex, age, location, income, educational level and energy intake.

Figure 1 showed differences in contribution of food groups to animal protein intake, between groups. Main sources of animal proteins were dairy products (28%) and fish (24%) in the Org–Med group while in other groups the main sources were dairy products followed by white meat. Conv–NoMed participants had the highest contribution of red meat.

### 3.4. Economic Indicators

Table 4 presents the economy-related indicators across the different groups of consumers. The daily cost of the diet was 8.59 €/day (95% CI = 8.55–8.63) in the Conv–NoMed group. The average cost of the Conv–Med diet was 9.11 €/day (95% CI = 9.03–9.19). However, the cost of the diet of subjects in the ‘organic’ groups was higher: 10.90 €/day (95% CI = 10.81–10.98) and 11.43 €/day (95% CI = 11.34–11.52) for the Org–NoMed group and for the Org–Med group respectively.

The cost of “100 organic calories” was not substantially different between organic food consumers and the other participants, while the cost of 100 conventional food calories increased along with the share of consumption of organic food in the diet. Portions of the total budget allocated to food were different between groups. Participants from the Org–Med group allocated a higher share of their budget (26.4% (95% CI = 26.0–26.8)) to food than other participants.

### 3.5. Diet-Related Sociocultural Indicators

Table 5 showed that organic food consumers (Org–NoMed and Org–Med) were more frequent to consider seasonality, product origin, production methods and ethical production as major purchase motives compared to the other consumers. Direct contact with producers and regional production were also considered as major factors for purchase, although to a lesser extent.

## 4. Discussion

The present study showed that indicators of sustainability related to health and nutrition, proxy of environmental impact and sustainability-related motivations for purchase and sociocultural aspects, were always better among Conv–Med, Org–NoMed or Org–Med participants than among Conv–NoMed participants. Furthermore, it is noteworthy that the combination of both Mediterranean diet and high organic food consumption (Org–Med group) was associated with better values of the sustainability-related indicators, apart from the cost of the diet.

### 4.1. Nutritional Aspects

Concerning indicators related to nutrition and health, the participants following a Mediterranean diet had better PANDiet and mPNNS-GS scores reflecting healthier food choices and a better adherence to the French nutritional recommendations. This was also expected since the Mediterranean dietary pattern shows some similarities with the French and other food-based guidelines (high consumption of fish, fruit and vegetables, moderate consumption of meat or moderate alcohol drinking). Thus, a study using optimization models has revealed that foods typical of the Mediterranean diet enable the achievement of overall nutrient adequacy [28]. Among Mediterranean diet followers, no substantial difference in terms of diet quality was observed according to organic food consumption.

In addition, it has been shown, in the NutriNet-Santé study in France [29] and in Germany [30], that regular organic food consumers exhibit a plant food-based diet and a better compliance with the guidelines for a healthy lifestyle. The results obtained herein are thus very consistent with these previous observations.

Participants following the Mediterranean diet also exhibited a higher diversity of the diet, as prescribed by the FAO: this is partly due to the high diversity of fruit and vegetables consumed. In contrast, participants of the Conv–NoMed group had the lowest diversity score and the highest energy density, reflecting a higher consumption of non-recommended foodstuffs (i.e., refined fats or sugars, alcohols and oils).

The Conv–NoMed group had, on average, the highest BMI while the groups of consumers with a high organic consumption or a high adherence to the Mediterranean diet showed comparable lower figures. Interestingly, the lowest BMI figure (by 2 points) was found in the Org–Med group. This is consistent with the findings reporting a lower BMI among organic foods consumers [29] or having a Mediterranean dietary pattern [31] and illustrates that participants combining both dietary patterns exhibited the lowest BMI. It is noteworthy that Org–Med participants were also more prone to have a high physical activity level, exhibiting therefore an overall healthier lifestyle.

To our knowledge, only one intervention study has combined Mediterranean diet and organic food consumption in its analysis. In that study, healthy individuals and chronic Kidney Disease patients had to consume a conventional diet followed by an organic Italian Mediterranean diet for 14 days. Several body and blood composition parameters were significantly different between the two diets (for example, fat mass was lower after 14 days of the organic diet, for both healthy and unhealthy participants) [32]. Although 14 days is a short period of time, such scant data suggest that combining Mediterranean diet with organic foods could be beneficial for health. 

### 4.2. Economic Aspects

The present data showed that switching to a more plant-based diet (i.e., Mediterranean diet) weakly affects the cost of the diet. Consistently, MacDiarmid et al. showed that a diet optimized to reduce meat intake compared to the British traditional diet exhibits similar cost than the average food expenditure in the United Kingdom [33]. A systematic review also reported that adopting Mediterranean dietary patterns was positively associated with increased costs of daily food consumption. However, some strategies, such as adopting less leafy greens or reducing energy density, could maintain an affordable cost for this diet [34].

Moreover, we observed that the cost of organic food consumption was higher, probably due to the higher prices of organic food compared to conventional [35]. These extra-costs may be explained by the production costs (especially labour costs) and the lack of structuration of organic agricultural sectors with a limited market share at present [35]. A better organization and technical innovations, driven by an expansion of consumers demand, could contribute to reducing the prices of organic food [35,36].

The share of the budget allocated to food was higher among organic consumers than conventional consumers. These results suggest that, probably for diet quality purposes, these subjects allocated a greatest share of their budget to food compared to other groups.

Interestingly, we found that the cost of 100 organic calories was similar between diet groups, while the cost of 100 conventional calories was higher for organic consumers. Several hypothesizes may be proposed to explain these findings. These consumers may pursue more expensive foods, such as meat, fish, or alcohol in the conventional market as these foods are difficult to find in the organic market [37].

### 4.3. Environmental Aspects

The environmental impact of dietary habits was estimated through two proxies: animal and plant proteins consumption and origin of the animal proteins consumed. Participants of the Conv–NoMed group exhibited the highest animal protein intakes. Moreover, they consumed more red meat whose production shows the most damaging environmental impacts [38,39] than other participants. Indeed, it is documented that livestock more considerably impacts the environment (higher greenhouse gas (GHG) emission, land use, water demand) compared to plant food production [38,39]. Conversely, participants of the Conv–Med and Org–Med groups had lower intakes of animal proteins than the other participants, suggesting a lower environmental impact of the Mediterranean dietary pattern. This finding can be interpreted in light of other studies. For example, a study concluded that if Mediterranean [2], pescetarian, vegetarian or vegan diets were largely adopted, they could offer substantial environmental benefits, as a marked decrease in greenhouse gas emissions [2,40]. However, another work highlighted that the effect of reducing meat consumption on GHG emission depended on the food substitutions made [41]. Moreover, organic farming seems to have a lower environmental impact than traditional conventional productions although this needs to be more precisely documented at the individual level [1,42,43]. For instance, this system improves soil fertility, biodiversity maintenance and protects natural resources [36]. High organic food consumption appears to be generally less harmful for the environment, although for some particular indicators and calculations per product unit, organic farming could be less efficient [42].

In turn, participants combining organic food in a Mediterranean diet may encompass two important parameters (farming system and high plant consumption) that contribute to the reduction of the global environmental impacts related to diet.

### 4.4. Sociocultural Aspects

Finally, participants of the Org–Med and Org–NoMed groups exhibited more often sustainable motivations and practices and seemed more prone to adopt alternative ways of consuming. This is in line with the observation that consumers with an ethical identity and those concerned about food safety are more likely to develop a positive attitude on organic food [43].

### 4.5. Limitations and Strengths

Some limitations of our study should be noted. First, caution is needed when extrapolating the results since the participants were volunteers involved in a long-term cohort focused on nutrition and health. For example, they were probably higher consumers of organic food than the general population. Secondly, concerning environmental impacts, only proxies were used; it could be of major interest to integrate objective indicators such as carbon water or nitrogen footprint, biodiversity or land use (as previously done in other works [44]) accounting for the mode of food production. Third, the cost of daily diet among certain subgroups may seem high compared to other studies. This is in part due to some methodological aspects that include the use of a FFQ, prone to misestimating food intake [45] similarly to other self-reported food consumption assessments, as well as the contribution of organic food to the diet which was particularly high and the specific socioeconomic profiles of the participants included in the analysis [14]. However, the portions of the budget allocated to food were relatively close to those evaluated in other studies [46]. Finally, when estimating nutrient intakes for the computation of dietary scores, the production method was not accounted for, while differences in food compositions between the organic and conventional markets have been reported [47,48]. For example, it was shown that organic bovine milk has a more desirable fatty acid composition than conventional bovine milk, along with higher α-tocopherol and iron contents, and lower iodine and selenium contents [49].

However, our study also exhibits important strengths. This study is the first to make a comparison at the individual level between two major potentially sustainable dietary pattern models. Moreover, our study is based on a large sample and a wide spectrum of accurate data, allowing the use of a large variety of indicators to assess the sustainability of the diet. Most of the indicators used were discussed during the proceedings of an International Workshop, organized by the FAO [50].

## 5. Conclusions

In conclusion, based on a population of volunteers exhibiting high organic food consumption, the results provide arguments showing that following a Mediterranean or organic diet may improve some indicators related to sustainability. Subjects following the Mediterranean dietary pattern with high organic food consumption were consumers who better fitted the definition of sustainability as prescribed by the FAO, except for economic considerations. Sustainable dietary patterns in their diversity (including food safety, modes of food production and sustainable forms of aquaculture) need to be taken into consideration in the ongoing transition towards sustainability. Further research should also give high priority to multidisciplinary methods and to the assessment of accurate sustainable indicators.

## Figures and Tables

**Table 1:** Description of the construction of the nutritional scores.

Name of the Score	Objective	Methods/Calculation	Score Components	Scale Range
Diet Quality Index Based on the Probability of Adequate Nutrient Intake PANDiet [21]	Reflects the adequacy between nutrient intakes and French recommendations for 24 nutrients	PANDiet is the average of two sub-scores. The adequacy sub-score is the average of the probability of adequacy * (between 0 and 1), multiplied by 100 for nutrients for which the usual intake should be above a reference value. The moderation sub-score is the average of the probability of adequacy * (between 0 and 1) for nutrients for which the usual intake should not exceed a reference value and penalty values.	Adequacy score: protein, total carbohydrate, total fat, poly-unsaturated fatty acids, fibre, vitamins A, B1, B2, B3, B6, B9, B12, C, D, E, calcium, magnesium, zinc, phosphorus, potassium and iron. Moderation score: protein, total fat, saturated fatty acids, carbohydrate, cholesterol, and sodium Penalty: retinol, niacin, vitamin B6, C, D, E, folate, calcium, magnesium, zinc, phosphorus and iron	0 to 100
mPNNS-GS [22]	Reflects the level of adherence to the French food-based recommendation defined by the PNNS (Programme National Nutrition Santé)	mPNNS-GS is the sum of components. We did not use the physical activity component.	Fruit and vegetables (0–2), starchy foods (0–1), whole-grain (0–1), dairy products (0–1), meat (0–1), seafood (0–1), added fat (0–1), sweets (−0.5–1), water and soda (0–1), alcohol (0–1), salt (−0.5–1.5), penalty if energy intake >105% of calculated energy needs	0 to 13.5
Literature-based adherence score of Mediterranean diet [8]	Assesses the adherence to the Mediterranean diet regardless of the sample	For each food group, three levels of consumption are considered (predefined portion) and 0, 1, or 2 points were given depending on the daily consumption and the component	Fruits, vegetables, legumes, fish, starches, meat, dairy products, alcohol and olive oil	0 to 18
Dietary diversity score [23]	Evaluates the number of food groups consumed per day	The score is the sum of individual food groups consumed on average per day, among 21 food groups. A minimum portion size is required for each food group to be included in the score: 40 g for vegetables or fruits, 15 g for grains, legumes, cheese and 30 g for other foods.	Grains, cereals, legumes, tubers, vitamin A-rich vegetables, dark green leafy vegetables, vitamin C-rich vegetables, soybeans and soy products, other vegetables, vitamin A-rich fruits, vitamin C-rich fruits, others fruits, organ meat, meat, eggs, seafood, fish, insects, dairy products, cheeses and fat	0 to 21

* Probability of adequate Nutrient Intake =F((x−r)(SDr2+SDy2)n), *F* was “probnorm” function in SAS, *x* was the mean intake, *SD_y_*^2^ was the day-to-day variability of intake, *n* was the number of days of dietary data, *r* was the nutrient reference value and *SD_r_*^2^ was the interindividual variability.

**Table 2:** Characteristics of the participants, *N* = 22,866, NutriNet-Santé study, 2014.

*N* (%)	Conv–NoMed	Conv–Med	Org–NoMed	Org–Med	*p* *
	*N* = 14,266 (62%)	*N* = 3498 (15%)	*N* = 2532 (11%)	*N* = 2570 (12%)	
**Mediterranean diet score (/18)**	7.55 (7.52–7.57)	11.81 (11.75–11.86)	8.39 (8.33–8.46)	12.31 (12.25–12.38)	<0.0001
**Contribution (in % of weight) of organic food to the diet**	15.5 (15.3–15.8)	22.4 (21.9–22.9)	68.2 (67.6–68.7)	74.6 (74.0–75.1)	<0.0001
**Age (years)**	52.9 (52.7–53.2)	57.0 (56.6–57.5)	53.8 (53.2–54.3)	54.6 (54.1–55.2)	<0.0001
**Male (%)**	27.07	31.45	21.80	22.45	<0.0001
**Educational level (%)**					
<High school diploma	22.89	22.38	20.62	17.55	<0.0001
High school	15.37	14.89	13.55	14.55	
Post-secondary graduate	61.75	62.72	65.84	67.90	
**Monthly income per household unit (%)**					
Refuse to declare	12.15	12.29	11.89	11.67	<0.0001
<1200 euros	11.21	9.35	9.12	10.86	
1200–1800 euros	22.21	19.13	22.24	21.13	
1800–2700 euros	25.33	25.44	26.78	26.50	
>2700 euros	29.09	33.79	29.98	29.84	
**Location (%)**					
Rural Community	21.61	19.70	25.24	24.82	<0.0001
Urban unit: population <20,000 inhabitants	15.51	14.47	16.23	14.82	
Urban unit: population between 20,000 and 200,000 inhabitants	18.16	17.38	17.77	18.44	
Urban unit: population >200,000 inhabitants	44.73	48.46	40.76	41.91	
**Type of diet (%)**					
Vegetarian diet	0.67	2.23	2.05	6.85	<0.0001
Vegan diet	0.21	0.77	1.22	4.75	<0.0001
**Smoking status (%)**					
Former smoker	39.25	44.54	40.09	41.56	<0.0001
Occasional smoker	3.38	2.92	3.59	2.96	
Current smoker	8.44	5.75	6.60	4.94	
Never smoker	48.93	46.80	49.72	50.54	
**Alcohol intake (%)**					
No or low drinker	5.05	4.69	5.96	7.12	<0.0001
Moderate drinker (<20 g/day for women and <30 g/day for men)	84.33	89.42	84.99	88.29	
High drinker	10.62	5.89	9.04	4.59	
**Physical activity (%)**					
Missing data	11.26	10.38	9.44	8.91	<0.0001
Low (<30 min/day)	21.77	14.35	17.81	12.76	
Medium (30–60 min/day)	36.59	37.54	37.01	38.05	
High (>60 min/day)	30.38	37.74	35.74	40.27	

Abbreviations: Conv–NoMed: Conventional consumers and non-Mediterranean diet followers; Conv–Med: Conventional consumers and Mediterranean diet followers; Org–NoMed: Organic consumers and non-Mediterranean diet followers; Org–Med: Organic consumers and Mediterranean diet followers; Values are % or means (95% CI); * *p* values referred to χ^2^ test or Kruskal–Wallis test as appropriate.

**Table 3:** Nutrition-related indicators, *N* = 22,866 NutriNet-Santé study, 2014.

	Conv–NoMed		Conv–Med		Org–NoMed		Org–Med	
	*N* = 14,266 (62%)		*N* = 3498 (15%)		*N* = 2532 (11%)		*N* = 2570 (12%)	
PANDiet * (/100)	63.71 ^a^	(63.57–63.84)	69.02 ^b^	(68.75–69.29)	64.72 ^c^	(64.41–65.03)	71.40 ^d^	(71.09–71.71)
mPNNS-GS * (/13.5)	8.19 ^a^	(8.17–8.22)	9.30 ^b^	(9.24–9.35)	8.45 ^c^	(8.39–8.51)	9.29 ^b^	(9.23–9.36)
Dietary diversity score * (/21)	8.73 ^a^	(8.70–8.76)	10.39 ^b^	(10.33–10.45)	9.24 ^c^	(9.16–9.30)	10.67 ^b^	(10.59–10.74)
Energy density ^†^ (kcal/100 g)	86.99 ^a^	(86.61–87.36)	84.00 ^b^	(83.29–84.72)	82.95 ^b,c^	(82.11–83.79)	82.38 ^c^	(81.54–83.21)
Total energy intake ^†^ (kcal/day)	2020 ^a^	(2010–2031)	2270 ^b^	(2249–2290)	1966 ^c^	(1942–1990)	2233 ^b^	(2209–2256)
Total protein intake ^‡^ (g/day)	94.37 ^a^	(94.05–94.69)	88.22 ^b^	(87.60–88.83)	88.90 ^b^	(88.18–89.61)	79.00 ^c^	(78.29–79.72)
Protein intake *^,§^ (%)	19.61 ^a^	(19.54–19.67)	18.17 ^b^	(18.05–18.29)	18.27 ^b^	(18.13–18.41)	16.43 ^c^	(16.29–16.57)
Animal Protein intake ^‡^ (g/day)	69.20 ^a^	(68.84–69.57)	55.32 ^b^	(54.61–56.03)	60.29 ^c^	(59.46–61.11)	39.01 ^d^	(38.18–39.83)
Plant Protein intake ^‡^ (g/day)	25.17 ^a^	(25.03–25.31)	32.89 ^b^	(32.62–33.16)	28.61 ^c^	(28.29–28.92)	40.00 ^d^	(39.68–40.31)
Plant/Animal protein intake ratio ^‡^	0.44 ^a^	(0.28–0.60)	0.66 ^a^	(0.35–0.98)	0.57 ^a^	(0.20–0.93)	1.38 ^b^	(1.01–1.74)
Body mass index (BMI) * (kg/m^2^)	24.89 ^a^	(24.81–24.96)	24.11 ^b^	(23.96–24.26)	24.09 ^b^	(23.92–24.27)	22.90 ^c^	(22.72–23.08)

Abbreviations: Conv–NoMed: Conventional consumers and non-Mediterranean diet followers; Conv–Med: Conventional consumers and Mediterranean diet followers; Org–NoMed: Organic consumers and non-Mediterranean diet followers; Org–Med: Organic consumers and Mediterranean diet followers; Values are adjusted means (95% CI). Means annotated with the same letter are not different (*p* > 0.05), using multiple testing adjustment with Bonferroni correction; * Adjustment for sex, age and energy intake; ^†^ Adjustment for sex and age; ^§^ Defined as the percentage of total energy intake.

**Table 4:** Economic-related indicators, *N* = 22,866, NutriNet-Santé study, 2014.

	Conv–NoMed		Conv–Med		Org–NoMed		Org–Med	
	*N* = 14,266 (62%)		*N* = 3498 (15%)		*N* = 2532 (11%)		*N* = 2570 (12%)	
Cost of the diet * (€/day)	8.59 ^a^	(8.55–8.63)	9.11 ^b^	(9.03–9.19)	10.90 ^c^	(10.81–10.98)	11.43 ^d^	(11.34–11.52)
Total organic energy intake ^†^ (kcal/day)	311 ^a^	(304–318)	526 ^b^	(513–539)	1186 ^c^	(1171–1202)	1566 ^d^	(1551–1582)
Cost of the intake for 100 organic calories ^†,‡^ (€)	0.58 ^a^	(0.56–0.59)	0.56 ^a^	(0.54–0.58)	0.58 ^a^	(0.55–0.60)	0.55 ^a^	(0.53–0.58)
Cost of the intake for 100 conventional calories ^†,§^ (€)	0.37 ^a^	(0.36–0.37)	0.39 ^b^	(0.38–0.40)	0.45 ^c^	(0.44–0.45)	0.54 ^d^	(0.53–0.55)
Portion of the total budget allocated to food ^||^ (%)	19.3 ^a^	(19.1–19.5)	22.0 ^b^	(21.7–22.3)	23.4 ^c^	(23.0–23.7)	26.4 ^d^	(26.0–26.8)

Abbreviations: Conv–NoMed: Conventional consumers and non-Mediterranean diet followers; Conv–Med: Conventional consumers and Mediterranean diet followers; Org–NoMed: Organic consumers and non-Mediterranean diet followers; Org–Med: Organic consumers and Mediterranean diet followers; Values are adjusted means (95% CI). Means annotated with the same letter are not different (*p* > 0.05), using multiple testing adjustment with Bonferroni correction; * Adjustment for sex, age and energy intake; ^†^ Adjustment for sex and age; ^‡^ Cost of the intake for 100 organic calories defined as the cost of total organic intake *100/energy intake from organic food (zero-calorie beverages are not included); ^§^ Cost of the intake for 100 conventional calories defined as the cost of total conventional intake*100/total energy from conventional food (zero-calorie beverages are not included); ^||^ Adjustment for sex, age and income.

**Table 5:** Motivations when purchasing foods, *N* = 22,818, NutriNet-Santé, 2014.

Purchase Motivations	Conv–NoMed		Conv–Med		Org–NoMed		Org–Med	
	*N* = 14,266 (62%)		*N* = 3498 (15%)		*N* = 2532 (11%)		*N* = 2570 (12%)	
**Seasonality**								
Marginal factor (%)	5.2 ^a^	(4.6–5.9)	3.6 ^b^	(2.8–4.4)	1.3 ^c^	(0.8–1.9)	1.2 ^c^	(0.7–1.8)
Medium factor (%)	13.8 ^a^	(12.8–14.8)	11.3 ^b^	(10.0–12.7)	5.4 ^c^	(4.3–6.4)	4.2 ^c^	(3.3–5.0)
Major factor (%)	81.0 ^a^	(79.8–82.1)	85.1 ^b^	(83.6–86.6)	93.3 ^c^	(92.2–94.5)	94.6 ^c^	(93.6–95.6)
**Product origin**								
Marginal factor (%)	7.9 ^a^	(7.1–8.7)	6.0 ^b^	(5.0–7.0)	1.5 ^c^	(1.0–2.1)	0.9 ^c^	(0.5–1.3)
Medium factor (%)	24.0 ^a^	(22.8–25.3)	21.2 ^b^	(19.5–23)	7.4 ^c^	(6.3–8.6)	9.0 ^c^	(7.7–10.2)
Major factor (%)	68.1 ^a^	(66.7–69.5)	72.7 ^b^	(70.9–74.6)	91.0 ^c^	(89.8–92.3)	90.2 ^c^	(88.8–91.5)
**Production methods**								
Marginal factor (%)	22.2 ^a^	(20.9–23.4)	13.8 ^b^	(12.4–15.2)	1.1 ^c^	(0.6–1.5)	0.2 ^d^	(0.0–0.4)
Medium factor (%)	39.9 ^a^	(38.4–41.3)	34.1 ^b^	(32.2–36.1)	7.9 ^c^	(6.8–9.1)	4.0 ^d^	(3.2–4.7)
Major factor (%)	38.0 ^a^	(36.5–39.4)	52.1 ^b^	(49.9–54.2)	91.0 ^c^	(89.8–92.2)	95.8 ^d^	(95.0–96.6)
**Ethical production**								
Marginal factor (%)	23.8 ^a^	(22.4–25.1)	15.7 ^b^	(14.2–17.2)	3.1 ^c^	(2.4–3.9)	2.2 ^d^	(1.6–2.8)
Medium factor (%)	42.5 ^a^	(41–43.9)	39.9 ^b^	(37.9–42.0)	18.3 ^c^	(16.5–20.0)	13. ^d^	(11.9–14.9)
Major factor (%)	33.8 ^a^	(32.4–35.1)	44.3 ^b^	(42.3–46.4)	78.6 ^c^	(76.8–80.5)	84.4 ^d^	(82.8–86.0)
**Direct contact with producers**								
Marginal factor (%)	27.0 ^a^	(25.6–28.3)	24.0 ^b^	(22.2–25.8)	13.4 ^c^	(12.0–14.9)	12.5 ^c^	(11.1–13.9)
Medium factor (%)	34.8 ^a^	(33.5–36.2)	33.6 ^b^	(31.7–35.6)	29.6 ^c^	(27.6–31.7)	29.1 ^c^	(27.0–31.2)
Major factor (%)	38.2 ^a^	(36.8–39.6)	42.4 ^b^	(40.3–44.4)	56.9 ^c^	(54.6–59.2)	58.4 ^c^	(56.1–60.7)
**Regional product**								
Marginal factor (%)	25.0 ^a^	(23.7–26.3)	22.5 ^b^	(20.7–24.2)	13.3 ^c^	(11.9–14.8)	14.0 ^c^	(12.5–15.5)
Medium factor (%)	34.9 ^a^	(33.5–36.2)	33.7 ^b^	(31.7–35.6)	28.4 ^c^	(26.3–30.4)	28.6 ^c^	(26.5–30.6)
Major factor (%)	40.1 ^a^	(38.7–41.5)	43.9 ^b^	(41.8–45.9)	58.3 ^c^	(56.0–60.6)	57.4 ^c^	(55.1–59.7)

Abbreviations: Conv–NoMed: Conventional consumers and non-Mediterranean diet followers; Conv–Med: Conventional consumers and Mediterranean diet followers; Org–NoMed: Organic consumers and non-Mediterranean diet followers; Org–Med: Organic consumers and Mediterranean diet followers; Values are % (95% CI) adjusted for sex, age, location, income and educational level. Means annotated with the same letter are not different (*p* > 0.05), using multiple testing adjustment with Bonferroni’s correction.
